# Severe Cardiovascular Effects of Prolonged Untreated Hyperthyroidism Manifesting As Thyroid Storm

**DOI:** 10.7759/cureus.26289

**Published:** 2022-06-24

**Authors:** Mohamed Hamed, Sarah Palumbo, Taaha Mendha

**Affiliations:** 1 Internal Medicine, Florida Atlantic University Charles E. Schmidt College of Medicine, Boca Raton, USA

**Keywords:** hyperthyroidism, cardiomyopathy, hyperthyroid cardiomyopathy, atrial flutter, thyroid-storm, thyrotoxicosis, heart arrythmia

## Abstract

A thyroid storm is a rare but life-threatening manifestation of thyrotoxicosis. It still remains a diagnostic challenge as there are no specific laboratory investigations or universally accepted criteria for diagnosing thyroid storms. Diagnosis is mainly based on clinical findings, evidence of hyperthyroidism, and life-threatening symptoms. A thyroid storm has a high risk of mortality mostly due to multi-organ failure and heart failure. Cardiovascular manifestations are the most common presentation of hyperthyroidism; cardiac involvement also has the potential to be the most serious complication. Management of cardiovascular manifestations should be managed aggressively to prevent long-term myocardial damage. A high index of suspicion should be maintained in young adults presenting with heart failure and arrhythmia. We present a case of potentially life-threatening cardiovascular effects of thyroid storm and management in the ICU.

## Introduction

A thyroid storm is a life-threatening, endocrinologic emergency that represents an extreme manifestation of acute thyrotoxicosis. In a prior United States survey, thyroid storm was diagnosed in 16.2% of thyrotoxicosis admissions [1]. The condition however carries a high risk of mortality of more than 10%, mostly from multi-organ failure and heart failure [2-4]. Cardiovascular manifestations remain the most common presentation of hyperthyroidism, and the most serious complications often occur as a result of cardiac involvement, which is often managed in an intensive care unit (ICU) setting [5].

## Case presentation

**Figure 1:**
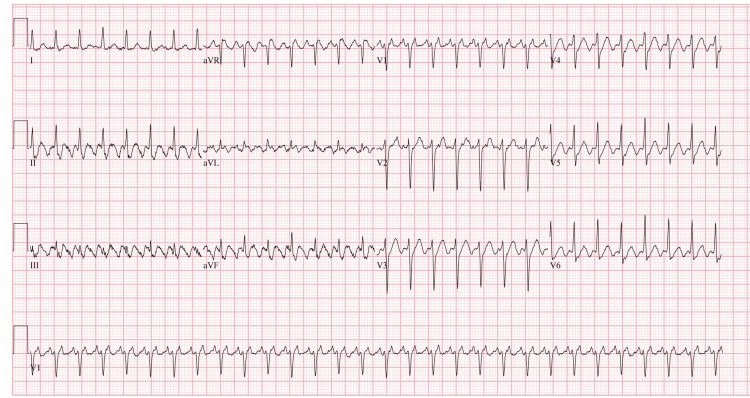
EKG on admission

**Figure 2:**
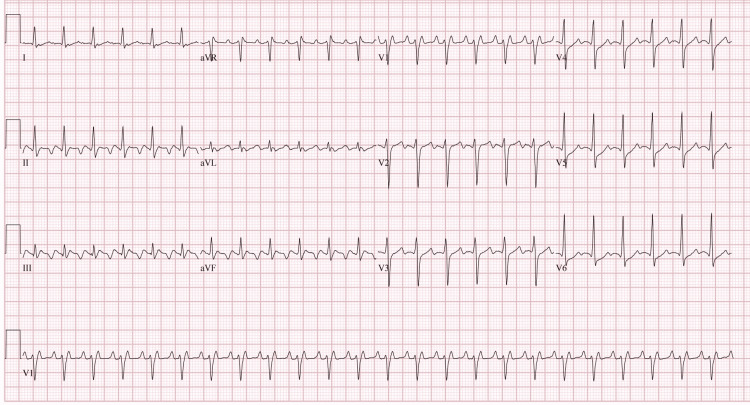
EKG after adenosine

**Figure 3:**
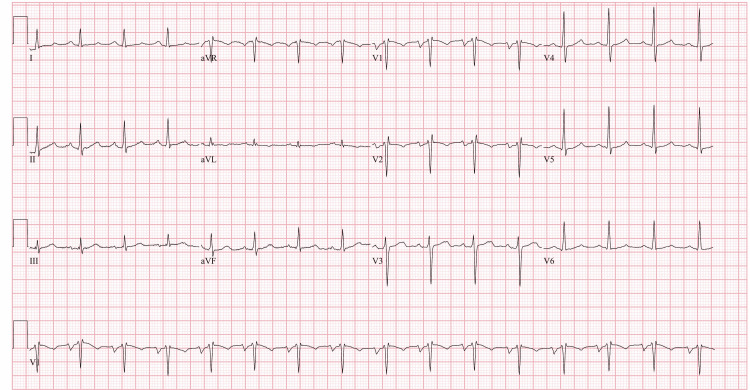
EKG after cardioversion

Our patient was a 43-year-old male who presented to the emergency department with a more than the two-month history of heat intolerance, palpitations, diaphoresis, tremors, nausea, diarrhea, and unintentional weight loss. Pertinent physical exam findings included tachycardia with regular heart rate up to 170 beats per minute, bilateral lid lag, pronounced stare, peripheral edema, and a diffusely enlarged, palpable thyroid. Additionally, the patient exhibited mild agitation. These findings were consistent with thyrotoxicosis. Utilizing the Burch-Wartofsky Point Scale, the patient’s score of 50 was highly suggestive of an impending thyroid storm. The patient was admitted to ICU with a diagnosis of thyrotoxicosis/thyroid storm confirmed by a thyroid-stimulating hormone (TSH) level of <0.005 and elevated T3 and free T4. A thyroid ultrasound demonstrated diffuse enlargement and hypervascularity indicating possible Graves Disease as the etiology for the patient’s thyrotoxicosis. This diagnosis was confirmed with thyroid-stimulating immunoglobulin (TSI) and anti-TSH receptor (anti-TSHr) antibodies. The patient was started on a regimen of propylthiouracil, esmolol, hydrocortisone, potassium iodide, and cholestyramine. Propranolol was added for persistent tachycardia in addition to its effects on the conversion of T4 to T3. Electrocardiogram (EKG) on admission showed supraventricular tachycardia (Figure 1). Adenosine was given for supraventricular tachycardia with a repeat EKG showing atrial flutter with 2:1 conduction (Figure 2). He was subsequently scheduled for cardioversion. His pre-cardioversion transesophageal echocardiogram showed a left ventricular ejection fraction (LVEF) of 15% and a severely dilated left ventricle. After cardioversion, his EKG showed normal sinus rhythm (Figure 3) and his EF improved to 25%-30%; however, severe mitral regurgitation was noted to persist despite the resolution of his arrhythmia. He remained inpatient for two additional days post-cardioversion. His symptoms resolved with additionally noted decreases in T3/T4 levels. Ultimately, he was discharged on methimazole and hydrocortisone in addition to lisinopril and carvedilol for his cardiomyopathy. He was to be evaluated for ablation therapy for atrial flutter prevention.

## Discussion

A thyroid storm is a rare manifestation of thyrotoxicosis with serious complications including heart failure and hypotension potentially resulting in cardiovascular collapse and death in young patients. This case illustrates the potential life-threatening cardiovascular effects of thyroid storms and the need for management in the ICU. A high index of suspicion should be maintained in young males presenting with heart failure and arrhythmia [6]. Although there are no universally accepted criteria for diagnosing thyroid storms, a scoring system using precise clinical criteria was introduced by Burch and Wartofsky for the identification of thyroid storms [7]. Diagnosis of thyroid storm is presently based on clinical findings, biochemical evidence of hyperthyroidism, and the presence of life-threatening symptoms including hyperpyrexia, cardiovascular dysfunction, and altered mentation [2]. This patient’s clinical status was concerning for adverse outcomes including cardiogenic shock in the setting of atrial tachyarrhythmia, severe mitral regurgitation, and tachycardia-induced cardiomyopathy. Supraventricular tachycardia is a potential risk factor for congestive cardiomyopathy and should be treated aggressively to prevent myocardial dysfunction [8]. Termination of arrhythmias can cause a significant reversal of cardiomyopathy with recovery in systolic function and reduction in chamber size [9-11]. Mortality rates as high as 17% associated with ICU-treated thyrotoxicosis have been documented in prior studies, which have seen the clinical associations between this condition and the development of heart failure and arrhythmias [12]. Additional analysis in retrospective studies noted statistically significant increases in survival rates for patients who were placed on beta-blockers as part of their treatment regimen; however, further studies are needed for a greater understanding of this condition [12]. The rapid and efficient assessment and treatment of thyroid storms has the potential to improve mortality and long-term outcomes for patients through both endocrine and cardiovascular interventions [9,11].

## Conclusions

Thyroid storm is a rare, life-threatening manifestation of thyrotoxicosis that requires close monitoring and management in the ICU. Cardiovascular effects of thyroid storm include diverse and important sources of potential mortality that should be treated aggressively to prevent long-term myocardial damage.
